# Application of short-term analysis of skin temperature variability in prediction of survival in patients with cirrhosis

**DOI:** 10.3389/fnetp.2023.1291491

**Published:** 2024-01-05

**Authors:** Noor-Ul-Hoda Abid, Travis Lum Cheng In, Matteo Bottaro, Xinran Shen, Iker Hernaez Sanz, Satoshi Yoshida, Chiara Formentin, Sara Montagnese, Ali R. Mani

**Affiliations:** ^1^ Network Physiology Laboratory, Division of Medicine, UCL, London, United Kingdom; ^2^ UCL Medical School, UCL, London, United Kingdom; ^3^ Department of Medicine, University of Padova, Padova, Italy; ^4^ Chronobiology Section, Faculty of Health and Medical Sciences, University of Surrey, Guildford, United Kingdom; ^5^ Institute for Liver and Digestive Health (ILDH), Division of Medicine, UCL, London, United Kingdom

**Keywords:** poincaré plot, prognosis, proximal temperature variability, time-series, liver failure

## Abstract

**Background:** Liver cirrhosis is a complex disorder, involving several different organ systems and physiological network disruption. Various physiological markers have been developed for survival modelling in patients with cirrhosis. Reduction in heart rate variability and skin temperature variability have been shown to predict mortality in cirrhosis, with the potential to aid clinical prognostication. We have recently reported that short-term skin temperature variability analysis can predict survival independently of the severity of liver failure in cirrhosis. However, in previous reports, 24-h skin temperature recordings were used, which are often not feasible in the context of routine clinical practice. The purpose of this study was to determine the shortest length of time from 24-h proximal temperature recordings that can accurately and independently predict 12-month survival post-recording in patients with cirrhosis.

**Methods:** Forty individuals diagnosed with cirrhosis participated in this study and wireless temperature sensors (iButtons) were used to record patients’ proximal skin temperature. From 24-h temperature recordings, different length of recordings (30 min, 1, 2, 3 and 6 h) were extracted sequentially for temperature variability analysis using the Extended Poincaré plot to quantify both short-term (SD1) and long-term (SD2) variability. These patients were then subsequently followed for a period of 12 months, during which data was gathered concerning any cases of mortality.

**Results:** Cirrhosis was associated with significantly decreased proximal skin temperature fluctuations among individuals who did not survive, across all durations of daytime temperature recordings lasting 1 hour or more. Survival analysis showcased 1-h daytime proximal skin temperature time-series to be significant predictors of survival in cirrhosis, whereby SD2, was found to be independent to the Model for End-Stage Liver Disease (MELD) score and thus, the extent of disease severity. As expected, longer durations of time-series were also predictors of mortality for the majority of the temperature variability indices.

**Conclusion:** Crucially, this study suggests that 1-h proximal skin temperature recordings are sufficient in length to accurately predict 12-month survival in patients with cirrhosis, independent from current prognostic indicators used in the clinic such as MELD.

## Introduction

Liver cirrhosis is recognized for its impact on the hepatic architecture, resulting in the development of chronic liver dysfunction. However, it also is indeed a multi-system disorder, involving several different organ systems, as it concomitantly disrupts the homeostasis of the circulatory, immune, and nervous systems. Thus, resulting in several systemic manifestations, pathognomonic of this disease, such as hepatic encephalopathy, portal hypertension, ascites, cardiomyopathy and hepatorenal syndrome, which consequently exacerbate disease severity and debilitate the quality of life exhibited in patients with decompensated disease (Huang et al., 2023).

Recent studies have revealed critical evidence for impaired organ system connectivity and responsiveness to physiological stimuli in patients with cirrhosis, both inherently and externally to environmental challenges (Figueiredo et al., 2012; Shirazi et al., 2013; Tan et al., 2020; Liu et al., 2022; Zhang et al., 2022). Thus, the assessment of organ system connectivity in patients with liver disease has two key benefits, firstly, it provides physiological insight into the complexity of the underlying pathophysiology (e.g., understanding the mechanism of autonomic dysfunction in cirrhosis). Secondly, it also yields crucial prognostic information which can be used chiefly during pivotal organ allocation protocols conducted prior to liver transplantation (Abid and Mani, 2022).

Physiological time-series analysis has extensively been used for the assessment of autonomic function in health and disease. Notably, reduced heart rate variability (HRV) has been shown to predict mortality in patients with cirrhosis (Mani et al., 2009; Bhogal et al., 2019; Jansen et al., 2019; Oyelade et al., 2021). However, HRV analysis does not hold the capacity to exhaustively predict survival in all patients with cirrhosis, predominantly due to patients exhibiting common arrythmias, such as atrial fibrillation as well as premature ventricular complexes (Oyelade et al., 2020). Hence, other physiological time-series have been investigated, whereby critical changes in the inherent variability of patient’s skin temperature recordings have been recently reported in patients with cirrhosis, to provide clinically useful prognostic information (Bottaro et al., 2020). This has also been reported in patients suffering from other critically ill conditions such as sepsis (Papaioannou et al., 2012; Papaioannou et al., 2013; Mani et al., 2018). Importantly, skin temperature fluctuations exhibit circadian changes, as well as short- and long-term fluctuations which can be assessed using different analytical and computational techniques based on non-linear dynamics, discussed further in Bottaro et al. (2020) Remarkably, proximal temperature variability (PTV) analysed from 24-h skin temperature recordings can predict mortality independent to the severity of liver cirrhosis, measured using MELD (Model for End Stage Liver Disease) or the Child-Pugh score currently used in the clinic (Bottaro et al., 2020).

Mechanistically, skin temperature fluctuations reflect both heat loss and heat gain and are hence influenced by thermoregulation, as well as the extent of skin perfusion which is regulated by the autonomic nervous system (Houben et al., 1994; Bottaro et al., 2020). Moreover, several lines of evidence have revealed that patients with cirrhosis suffer from autonomic dysfunction and impaired vascular responsiveness to adrenergic stimuli (Dümcke and Møller, 2008). Thus, justifying the notion that it is indeed reasonable to assess skin temperature and its inherent variability to determine the extent of autonomic dysfunction in patients with cirrhosis. This is further reinforced by the study by Garrido et al. whereby significant changes were documented in patients’ distal and proximal temperature gradients in comparison to healthy individuals (Garrido et al., 2017). Temperature variability analysis features both short- and long-term variability, whereby the former represents minute-to-minute changes of skin perfusion, and the latter is largely illustrative of circadian variation. We have recently reported that, while both short-term and long-term PTV indices could predict survival in patients with cirrhosis, only measures of short-term PTV were shown to be independent of the severity of liver failure in predicting survival (Bottaro et al., 2020). In this study, 24-h proximal temperature recordings were used. However, these are often not feasible in the context of routine clinical practice, and so, this present study aimed to determine the shortest length of time that can accurately provide such clinically useful prognostic information. Hence, in this study we used data recorded from our previous study Bottaro et al., to further analyse the prognostic value of shorter proximal temperature time-series, which are indeed more clinically feasible to record in patients with cirrhosis for future potential clinical application.

## Methods

*Ethics statement*: The study was approved by the ethics committee at the University Hospital of Padova (4196/A0/17) and adhered to the Good Clinical Medicine and the Declaration of Helsinki guidelines. Written informed consent was provided by all patients involved and anonymised data was collected and stored appropriately.

*Patients:* As stated in the original study, a total of 40 individuals diagnosed with cirrhosis, who were admitted to Medical Clinic 5 at the University Hospital of Padova, participated in this study (Bottaro et al., 2020; Oyelade et al., 2020). The study took place between 6 April 2017 and 2 February 2019 (date of the first temperature recording-last survey date after follow-up for survival analysis). The severity of cirrhosis was assessed using the application of the Model for End-Stage Liver Disease (MELD) scoring systems. Exclusion criteria were applied to individuals who were below 16 years of age, patients with cirrhosis following a liver transplant, those with a significant co-morbidity, febrile disease, history of neurological or psychiatric disorders unrelated to hepatic encephalopathy, any ongoing alcohol misuse, or those currently using psychoactive medications. Patients were followed up for 12 months, during which data pertaining to any instances of mortality or liver transplantation were recorded, as described in the original study (Bottaro et al., 2020; Oyelade et al., 2020).

*Proximal skin temperature recording:* Three wireless temperature probes (iButtons, model DS1922L-F5, Maxim Integrated, CA, United States of America) were positioned on the patients’ bodies with a sampling rate of 1 recording per 3 min, as described by Bottaro et al. (2020). In brief, the probes were placed on the abdomen, intra-clavicular area, and mid-thigh areas to estimate the proximal body temperature fluctuations. Proximal temperature time-series were generated using a weighted average of the three proximal sensors, in accordance with the methodology outlined by Longato et al. (2017) using the following formula: T _proximal_ = 0.379 x T _abdomen_ + 0.262 x T _intra-clavicular_ + 0.359 x T _mid-thigh_.

The duration of temperature monitoring lasted for a continuous 24-h period; Daytime (awake) and nighttime (asleep) data were then determined for each participant based on a sleep diary.

**FIGURE 1:**
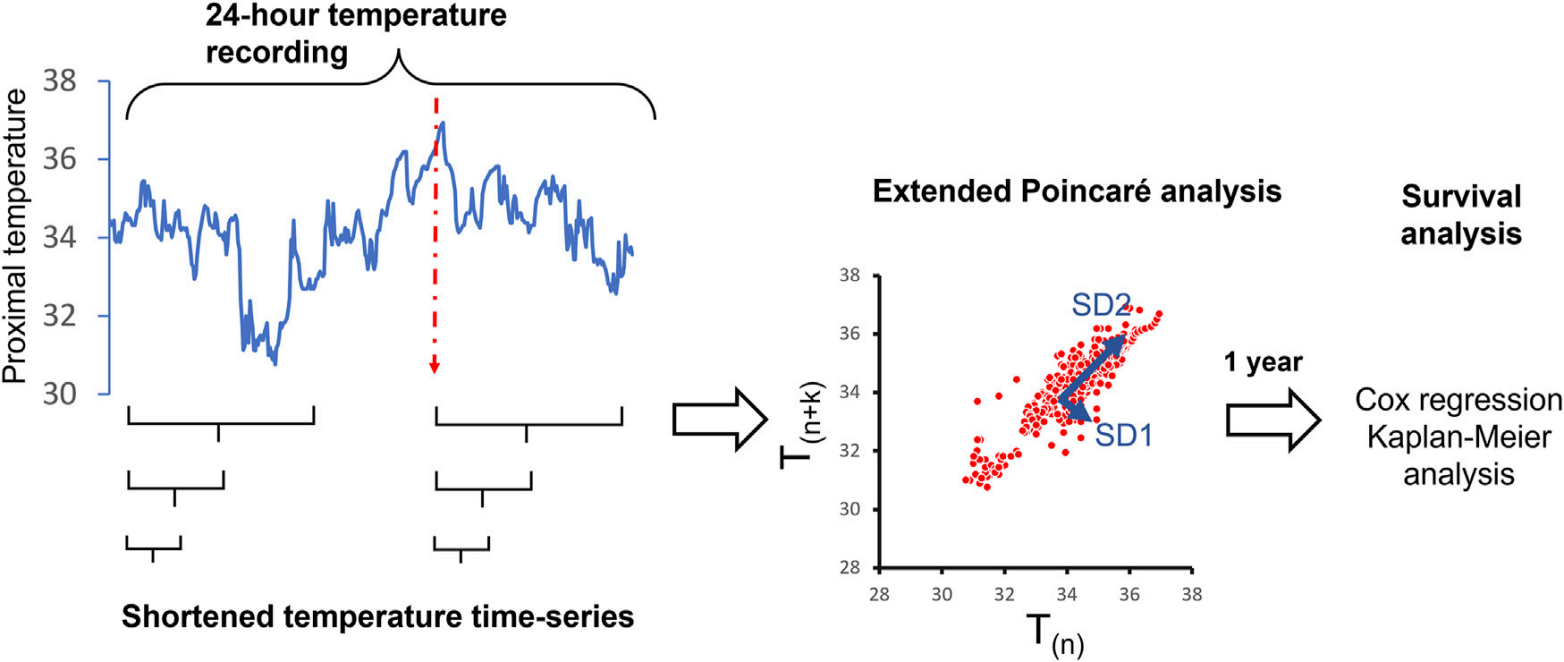
Overview of the study methodology to determine the shortest length of time from 24-h proximal temperature recordings, that can predict 12-month survival post-recording in patients with cirrhosis. The red dotted line indicated the initiation of the patient’s sleeping time.

*Temperature variability analysis:* From 24-h temperature recordings, different lengths of recordings (e.g., 30 min, 1, 2, 3 h) were extracted sequentially for temperature variability analysis (Figure 1). The Extended Poincaré plot was used to calculate proximal temperature variability (PTV) at different lags, as described by Satti et al. (2019). In brief, the Extended Poincaré plot analyses the correlation existing between consecutive data points within a time-series. This analysis involves comparing data points at different positions in the series, denoted as T_(n)_ and T_(n+k)_. Here, ‘T’ represents skin temperature and ‘k' (referred to as the ‘lag’) represents a discrete time interval along the time-series, and it is not limited to adjacent points, as seen in the traditional Poincaré plot. In the Poincaré plot, the standard deviation that is perpendicular to the line of identity is used to measure the short-term variability observed in the physiological time-series and is hence denoted as SD1 (Standard Deviation 1). SD1 is considered a measure of short-term variability in temperature time-series because it primarily reflects the minute-to-minute variations or fluctuations in the temperature signals, which represent the time between consecutive temperature recordings. In Poincaré plots, SD1 is calculated as the standard deviation of the points perpendicular to the line of identity, which connects consecutive temperature signals. On the other hand, the standard deviation that runs parallel to the line of identity in the Extended Poincaré plot is referred to as SD2 (Standard Deviation 2) and quantifies the long-term variability exhibited in the physiological time-series (Mani et al., 2009). In essence, SD2 captures a broader spectrum of variability, including long-term components. In this study, SD1 and SD2 were calculated using a code in MATLAB as described in Satti et al., 2019.

*Statistical analysis:* Short-term (SD1) and long-term (SD2) PTV indices of survivors and non-survivors were then compared using either *t*-test or the nonparametric Mann–Whitney *U*-test according to the distribution of the data. Univariate or bivariate Cox regression was used for survival analysis. *p*-values less than 0.05 were considered statistically significant. The Receiver Operating Characteristic (ROC) curve was employed to identify optimal cutoff points (the Youden’s index) that offer the highest levels of sensitivity and specificity in predicting survival. These cutoff points were then utilized to categorize patients on the Kaplan-Meier graph, with their placements based on their respective PTV indices.

## Results

This study extends the analysis of our previous report (Bottaro et al., 2020) to determine the shortest duration of proximal skin temperature recording that can predict survival in a cohort of patients with cirrhosis. The demographics of the study population and mortality rate is presented in the original study (Bottaro et al., 2020; Oyelade et al., 2020). In brief, there were 30 male and 10 female participants in this cohort. During the follow-up period: 23 survived and 17 mortality events were registered. There were no significant differences in age and gender between the survivor and non-survivor groups. Non-survivors exhibited a greater degree of liver dysfunction according to the MELD score (17.8 ± 1.8 *versus* 23.8 ± 2.0 in survivors *versus* non-survivors, *p* < 0.05).

Comparison of mean proximal temperature and total PTV between survivors and non-survivors: The mean proximal temperature in 24-h recordings was 35.05 ± 0.16 and 35.36°C ± 0.19°C in survivors and non-survivors, respectively (*p* = 0.22). The total proximal temperature variability (PTV), as assessed by measuring the standard deviation of proximal time-series in 24-h recordings, was similar in both groups (0.674 ± 0.071 *versus* 0.504 ± 0.045, *p* = 0.065). Overall, there was no significant difference in the mean proximal temperature when the time-series were broken down into shorter pieces. Details of mean and total PTV in shorter time-series are shown in Supplementary Material (Supplementary Appendix S1 and S2).

*Comparison of PTV indices between survivors and non-survivors:* The indices included in the PTV analysis were SD1 and SD2 for lag (k) values from 1 to 10 in the Extended Poincaré plot. This analysis was performed for 30 min, 1, 2, 3 and 6 h during the awake period in the day and 1, 2, 3 h during the sleep phase at night. The 24-h PTV indices did exhibit significant differences between survivors and non-survivors for SD1, when k was from 2 to 10 (Bottaro et al., 2020). The results of SD1 and SD2 for shorter time durations are shown in Supplementary Material (Supplementary Appendix S1). Notably, the 6-h daytime PTV analysis showed significance for SD1, when k was from 3 to 10 and for SD2, all k-values. Both the 3-h and 2-h daytime PTV indices, i.e., SD2 was significant for all k-values. Critically, the 1-h daytime PTV indices revealed SD2 to be significant, when k = 2–4. However, the 30-min daytime PTV indices were not significant for any of the parameters. In the sleep phase at night Supplementary Material (Supplementary Appendix S2). The 3-h PTV indices showed significance for SD1 for most lags (k-values), whilst the 2-h PTV indices revealed significance of SD1, when k was higher than 5. However, the 1-h PTV indices did not show any significant differences between survivors and non-survivors Supplementary Material (Supplementary Appendix S2).

*Correlation between PTV indices between 1-h and 24-h time-series recording*: As shown in Table 2, SD1s (at different lags) were significantly correlated between 1-h and 24-h time-series. Furthermore, there was a significant correlation between SD1s calculated from 24-h recordings and SD2s calculated from 1-h recordings. This indicates that the physiological meaning of SD1 and SD2 and thus, short- and long-term PTV, respectively, changes with the duration of the recording (i.e., the long-term fluctuations in 1-h recordings are correlated with the short-term variability in 24-h recordings).

*Survival analysis:* Cox regression analysis on significant parameters obtained from Supplementary Material (Supplementary Appendix S1 and S2) revealed that several PTV indices were significant predictors of survival. In the 24-h time-series, our previous report showed that only SD1 (k = 1, 2 and 3) could predict survival in this cohort. The 6-h indices showed significance for all SD1 values and SD2 when k = 1, 2 and 3. In shorter time-series, the significant PTV indices transitioned from SD1 being predictive to SD2 as the sole significant index. As shown in Supplementary Material (Supplementary Appendix S3-A4), in the 1-h time-series during the awake phase, the hazard ratio for SD2 (when k = 2, 3 and 4) were significantly below 1, which indicated that higher SD2 values were associated with lower mortality (higher survival).

Bivariate Cox regression analysis on parameters that were predictors of survival revealed that some PTV indices predicted survival, independently of the MELD score Supplementary Material (Supplementary Appendix S3B). In the awake phase, the 1-h indices were significant for SD2, when k = 2 and 3 Table 1. The 2-h indices only showed significance for SD2 when k was 1, 2, 3 and 4. In the asleep phase, the 2-h and 3-h PTV indices revealed significance for SD1, in some lags (k), and were independent predictors of survival from the MELD score Supplementary Material (Supplementary Appendix S4).

**TABLE 1:** Correlation between SD1 and SD2 at different lag (k) between 1-hour (horizontal axis) and 24-hours (vertical axis) proximal temperature variability analysis. The correlation coefficient was calculated using Pearson’s correlation coefficient (r). Coefficients that are not statistically significant are represented by the value zero.


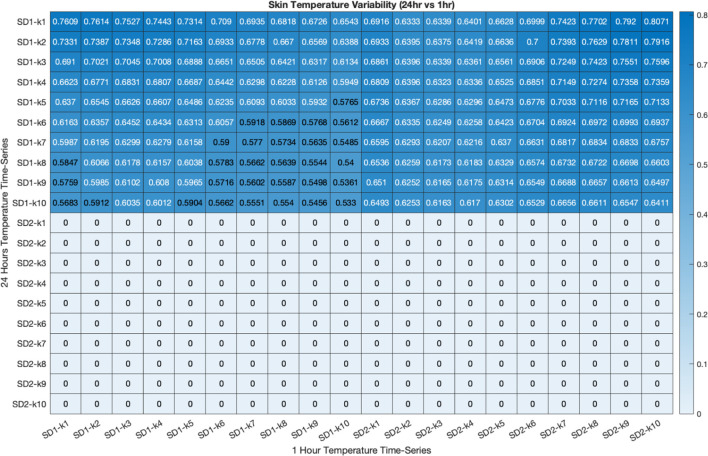

**FIGURE 2:**
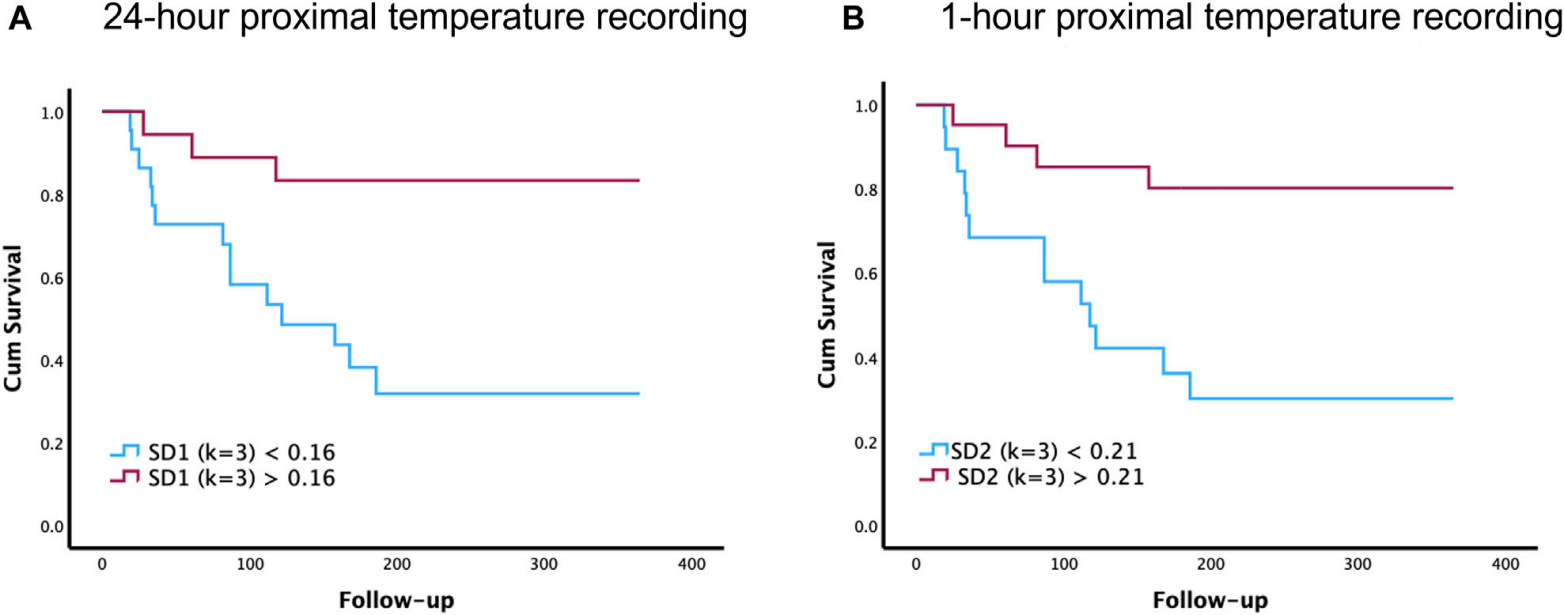
Comparison of 1-h and 24-h proximal temperature variability (PTV) analysis on survival in patients with cirrhosis. The Kaplan–Meier survival graphs below illustrate how PTV indices can predict survival in patients with cirrhosis. Specifically, these survival graphs depict the overall survival of patients with cirrhosis, above and below the cutoff value of SD1 (k = 3) for 24-h recordings **(A)** and SD2 (k = 3) for 1-h recordings **(B)**. Follow-up time is recorded in days after the primary proximal temperature recordings taken at the start of the study.

Figure 2 illustrates the Kaplan-Meier graphs for proximal temperature variability (PTV) indices extracted from 1-h and 24-h time-series recordings. These graphs show that PTV calculated from both 1-h (SD2) and 24-h (SD1) can predict survival in patients with cirrhosis. Log-rank test, Chi square = 7.33, *p* = 0.007 for 24-h and log-rank test, Chi square = 7.36, *p* = 0.007 for 1-h.

Since SD2 calculated from the first 1-h recorded in the proximal temperature time-series could predict 1-year survival in our cohort, we then aimed to investigate if any 1-h temperature recording extracted from daytime data collected could be used to predict survival. Thus, in a sub-study, a set of 1-h recordings was randomly extracted from the daytime period of each 24-h temperature time-series, and SD2 (k = 3) was then calculated for each 1-h time-series. This process was then repeated 100 times, and the hazard ratio was calculated for predicting mortality. The results of this analysis showed that the mean estimated hazard ratio (95% confidence interval) for randomly selected 1-h temperature time-series was 0.0579 (0.0436–0.0721), indicating its significant predictive value (*p* < 0.001). The same analysis was then applied to assess the area under the curve (AUC) for the ROC curves following 100 iterations, in calculating the AUC for predicting survival in any of the randomly selected 1-h signals from the daytime temperature time-series. The results showed that the mean AUC (95% confidence interval) for any randomly selected 1-h temperature time-series to predict survival was 0.66 (0.58–0.74), again indicating significant predictive value (*p* < 0.001).

## Discussion

In general, the purpose of this study was to determine the shortest length of time from 24-h proximal temperature recordings, that can accurately and independently predict 12-month survival post-recordings in patients with cirrhosis, especially during both daytime and night-time hours. Indeed, 1-h was found to be significant during daytime hours, whereby SD2 (k = 3 and 4), was found to be independent to MELD and thus the extent of disease severity. As expected, longer durations of time were also predictors of mortality for most PTV indices as shown in Supplementary Material (Supplementary Appendix S1). Based on these results, we wondered if only the first hour of each 24-h temperature recording is significant in predicting mortality. Thus, random selection was then conducted to choose any randomly selected 1-h length temperature sections from each daytime recording for subsequent PTV analysis, using the Extended Poincaré plot. This process was then repeated several times and the results revealed that the estimated hazard ratio was statistically less than 1, indicating significant prognostic value. Of note, the first hour of each temperature time-series recording before random data extraction was always statistically significant in the Cox regression analysis, suggesting its potential use in clinical practice. During night-time hours however, the minimum duration of time that could predict mortality was 2 h, whereby PTV indices were independent to MELD. Longer durations were also significant, of which most SD1 indices held independent prognostic capacity.

Crucially, in 24-h temperature-recordings, SD1 is indeed the short-term PTV, likewise in 1-h recordings, whereas SD2 is indeed the long-term PTV in both 24 h and 1-h recordings. However, the long-term PTV (SD2) of the shortened 1-h time-series is physiologically equivalent to the short-term PTV (SD1) of the 24-h time-series in predicting mortality. Hence, revealing how the physiological meaning of SD1 and SD2 and thus short- and long-term PTV, respectively, changes with the duration of the recording (Table 2). This is since long-term variability, SD2, reflects circadian changes in 24-h recordings, whereas SD2 in 1-h temperature recordings is reflective of a multitude of factors (e.g., skin perfusion), all giving rise to the short-term variability exhibited in skin temperature and thus thermoregulatory control. This also holds true with regards to the longer time-series during night-time hours, in which SD1 was significant, in comparison to the shortened 1-h daylight time-series, in which SD2 and hence long-term TV was significant. These findings are hence in line with our previous original study, whereby short-term PTV calculated from 24-h time-series was significantly independent to current prognostic indicators (MELD), and thus the extent of liver dysfunction (Bottaro et al., 2020). In general, shortened PTV indices in this study held prognostic value, independently of the MELD score. Thus, reinforcing the potential of utilising physio-markers, such as PTV analysis in routine clinical practice.

**TABLE 2:** Predictive effect of proximal temperature variability (PTV) indices extracted from 1-hour temperature time-series data on one-year mortality. Bivariate Cox regression analysis was used to assess the independence of PTV parameters from markers of liver failure (MELD) in predicting mortality.

Variable	β	Hazard ratio	95% Confidence Interval	p-value
SD2 (k=2)	-4.082	0.017	0.000 – 0.808	**0.039**
MELD	0.067	1.069	1.010 – 1.131	**0.021**
SD2 (k=3)	-4.298	0.014	0.000 – 0.919	**0.046**
MELD	0.065	1.067	1.009 – 1.130	**0.024**
SD2 (k=4)	-4.419	0.012	0.000 – 1.064	0.053
MELD	0.065	1.067	1.009 – 1.130	**0.023**

β is the coefficient of Cox regression analysis., Hazard ratio = Exp (β) = e^β^. The level of significance was set at p < 0.05 (bold values).

Given that a much shorter skin temperature recording can predict survival in patients with cirrhosis, it is indeed much more advantageous than the initially reported 24-h PTV indices for applications such as liver transplant allocation, as it minimises the likelihood of electrode detachment. This is since the patient’s daily activities, movement or indeed discomfort can interfere with the temperature sensors tethered to the skin and thus affect the accuracy of the temperature recording. Furthermore, a shorter duration of PTV measurement encourages patient compliance, whereby the attachment of temperature sensors at different skin regions to evaluate their disease prognosis can be easily conducted in an outpatient visit. Thus, the application of body temperature variability analysis can potentially be used in clinical practice as a novel pre-transplant screening tool to stratify patients awaiting liver transplantation to different priority groups. While these results are indeed interesting, it is nonetheless important to investigate in future studies whether PTV indices can potentially act as co-morbidity factors when considering patients for liver transplantation or can act as true prognostic indicators (Bhogal et al., 2018). Moreover, PTV analysis alongside the MELD score, provides additional useful information regarding survival outcomes, and its non-invasive evaluation of thermoregulation in patients may therefore be valuable in assessing the broader manifestations exhibited in cirrhosis. In fact, the addition of physiological markers including HRV, EEG or the Parenclitic network indices to the MELD score have demonstrated better prognostic capability than MELD score alone in previous studies (Montagnese et al., 2015; Bhogal et al., 2019; Abid and Mani, 2022, Zhang et al., 2022). Recent studies have consistently shown that a network physiology approach to complex diseases provide additional useful information which is often not detected by traditional prognostic indices (Oyelade et al., 2023). Thus, future clinical studies await the application of physio-markers such as HRV and PTV analysis in the clinical management of patients with chronic liver failure. PTV analysis holds the advantage that it can be conducted even in patients with common arrhythmias, such as atrial fibrillation, etc (Oyelade et al., 2020). Signal processing of proximal temperature data is also methodically easier, as there is no need for high frequency ECG recordings or detection of inter-beat intervals which requires additional algorithm and imputations. In this study, temperature recordings were done with a sampling rate of 1 recording per 3 min, which is much lower than the conventional sampling rate (256 Hz) required for HRV analysis.

Since the dynamics of fluctuations in physiological signals exhibit long-term correlation and memory (Shirazi et al., 2013; Mazloom et al., 2014; Taghipour et al., 2016), we used the Extended Poincaré method for the analysis of proximal temperature variability (PTV). This method measures the autocorrelation in physiological time-series and is able to quantify both short-term and long-term variability, which can hence be interpreted physiologically (Satti et al., 2019). We then wondered whether alternative non-linear approaches could be applied to analyse PTV in shortened time-series within our cohort. Subsequently, we employed Multiscale Poincaré analysis, as outlined by Henriques et al. (2016). The results of this analysis also showcased that 1-h daytime proximal temperature time-series were significant predictors of survival in cirrhosis, and were found to be independent to the MELD score and thus the extent of disease severity (data not shown). This indicates that simple computational methods can be utilised for PTV analysis, and the results remain significant even when alternative analytic methods are employed. In addition, we did not incorporate entropy measures for the analysis of shortened time-series due to the low data recording resolution (1 signal per 3 min), which thus did not permit us to estimate entropy effectively within the limited data points in the shortened time-series.

Our results indicate that reduced short-term PTV is associated with a poor outcome in patients with cirrhosis. Short-term skin temperature fluctuation reflects the dynamic interplay between heat loss and heat gain at a minute-by-minute time scale. Since the dynamics of skin perfusion play an important role in short-term skin temperature fluctuation, the reduced PTV in non-survivors might be linked to impaired control of peripheral vascular contraction in patients with cirrhosis. In fact, it is well-known that advanced cirrhosis is associated with significant impaired vascular responsiveness to sympathetic stimulation. Several mechanisms have been shown to be responsible for the blunted responsiveness to vasoconstrictors in advanced cirrhosis (Dümcke and Møller, 2008). It appears that both shear stress in response to portal hypertension and endotoxemia due to portocaval shunt stimulate the release of vasodilators such as nitric oxide, which reduces noradrenaline-induced vasodilation in cirrhosis (Iwakiri and Kim, 2015; Abid and Mani, 2022). This, at least in theory, can impair rhythmic oscillatory vasomotion in the skin in patients with severe physiological network disruption and explain the reduced short-term PTV in non-survivors with liver cirrhosis. In this study, we did not directly measure cutaneous vasomotion, and thus, this hypothetical mechanism remains to be further investigated in the future. A reduction in skin temperature variability is not only observed in cirrhotic patients with a poor outcome, but it has also been reported that reduced skin variability is associated with future disease risk in an otherwise healthy population (Brooks et al., 2023). Thus, reduced temperature variability may not be a liver disease-specific phenomenon and may be considered a signature for thermoregulatory network disruption in various systemic diseases, such as cirrhosis (Bottaro et al., 2020) and sepsis (Papaioannou et al., 2012; 2013).

In the present study, while PTV calculated from 1-h daytime temperature recordings provided sufficient information for predicting survival in patients with cirrhosis, nighttime temperature time-series required a longer duration to be used as a prognostic indicator. We did not investigate the reason behind this difference in day and night PTV dynamics. However, it is well-known that core body temperature drops to the nadir value during the acrophase at night. This change in body temperature can be detected in the proximal skin temperature signals as well as in distal-proximal temperature gradients (Garrido et al., 2017). These circadian alterations in temperature play a role in the modulation of sleep/arousal (Kräuchi and Wirz-Justice, 2001; Dvir et al., 2018) and might be mechanistically involved in the synchronization of peripheral clocks with the central circadian clocks (Brown et al., 2002). Nevertheless, the nighttime changes in body temperature make the dynamics of fluctuations in proximal temperature more complex than during the daytime. Short-term PTV at night might have more non-vascular components, and thus, a longer duration of temperature recording might be needed for predicting survival in patients with chronic liver failure. Testing such speculative hypotheses remains to be investigated in future studies.

One of the limitations of this study was its relatively small sample size and that it also was not a multi-centre heterogenous study. Core body temperature dynamics were also not recorded, due to clinical unfeasibility and their requirement for telemetric probes. Hence, proximal temperature variability analysis was used, however, the limitation of these measurements is that they do not provide precise information about the myriad of factors that contribute to a single reading, namely, the rate of blood-flow in the cutaneous circulation, as well as the sympathetic innervation regulating the vascular tone, core body temperature and also the degree of subcutaneous adipose tissue situated locally below the iButton. Nonetheless, PTV analysis does represent a more physiologically representative depiction of skin perfusion than core body temperature. Moreover, the use of computational analysis of physiological time-series, does indeed simplify the inherent complex dynamics of a physiological system for its application in medicine. Thus, in the future, a further longitudinal study employing larger cohort will further validate the true prognostic capacity of 1-h daytime PTV indices in patients with cirrhosis.

To conclude, this study has revealed the shortest duration required of a proximal skin temperature recording that can accurately predict 12-month survival in patients with cirrhosis, independent to current prognostic indicators such as MELD. Crucially, this finding has potential applicability in clinical practice by increasing the accuracy of organ allocation and thus selecting the patients most in need of liver transplantation.

## Data Availability

The raw data supporting the conclusion of this article will be made available by the authors, without undue reservation.
