# Supplementary material for: Application of short-term analysis of skin temperature variability in prediction of survival in patients with cirrhosis
Source: Front Netw Physiol. 2024 Jan 5;3:1291491. doi: 10.3389/fnetp.2023.1291491 (PMC10796461; doi:10.3389/fnetp.2023.1291491)
Supplement: Supplementary file 1 [file DataSheet1.pdf]

Supplementary materials

Appendix 1. Comparison between proximal skin temperature variability indices between survivors and non-survivors at different time-series lengths (30-minute to 6-hour) during awake phase.

A. 6-hour length time-series

	Survivors	Non-survivors	p-value
**Number**	23	17	-
**Mean proximal temperature (°C)**	35.28 ± 0.18	35.45 ± 0.24	0.468
**SD of proximal temperature (°C)**	0.483 ± 0.041	0.341 ± 0.036	**0.017**
**SD1 (k = 1) (°C)**	0.081 ± 0.006	0.065 ± 0.008	0.098
**SD1 (k = 2) (°C)**	0.141 ± 0.010	0.111 ± 0.013	0.070
**SD1 (k = 3) (°C)**	0.183 ± 0.013	0.139 ± 0.015	**0.034**
**SD1 (k = 4) (°C)**	0.214 ± 0.015	0.159 ± 0.017	**0.023**
**SD1 (k = 5) (°C)**	0.238 ± 0.017	0.174 ± 0.018	**0.017**
**SD1 (k = 6) (°C)**	0.258 ± 0.019	0.186 ± 0.020	**0.015**
**SD1 (k = 7) (°C)**	0.275 ± 0.021	0.196 ± 0.021	**0.013**
**SD1 (k = 8) (°C)**	0.289 ± 0.023	0.204 ± 0.022	**0.014**
**SD1 (k = 9) (°C)**	0.304 ± 0.025	0.212 ± 0.024	**0.014**
**SD1 (k = 10) (°C)**	0.317 ± 0.026	0.220 ± 0.026	**0.013**
**SD2 (k = 1) (°C)**	0.673 ± 0.058	0.472 ± 0.049	**0.017**
**SD2 (k = 2) (°C)**	0.659 ± 0.059	0.460 ± 0.049	**0.018**
**SD2 (k = 3) (°C)**	0.646 ± 0.059	0.451 ± 0.048	**0.020**
**SD2 (k = 4) (°C)**	0.634 ± 0.059	0.443 ± 0.048	**0.022**
**SD2 (k = 5) (°C)**	0.623 ± 0.058	0.436 ± 0.048	**0.024**
**SD2 (k = 6) (°C)**	0.612 ± 0.058	0.431 ± 0.047	**0.027**
**SD2 (k = 7) (°C)**	0.601 ± 0.058	0.425 ± 0.047	**0.031**
**SD2 (k = 8) (°C)**	0.591 ± 0.057	0.420 ± 0.046	**0.033**
**SD2 (k = 9) (°C)**	0.580 ± 0.057	0.413 ± 0.046	**0.036**
**SD2 (k = 10) (°C)**	0.570 ± 0.056	0.407 ± 0.045	**0.039**

B. 3-hour length time-series

	Survivors	Non-survivors	p-value
**Number**	23	17	-
**Mean proximal temperature (°C)**	35.11 ± 0.16	35.31 ± 0.25	0.486
**SD of proximal temperature (°C)**	0.386 ± 0.039	0.247 ± 0.033	**0.014**
**SD1 (k = 1) (°C)**	0.077 ± 0.008	0.065 ± 0.010	0.375
**SD1 (k = 2) (°C)**	0.132 ± 0.015	0.111 ± 0.017	0.331
**SD1 (k = 3) (°C)**	0.171 ± 0.018	0.138 ± 0.019	0.215
**SD1 (k = 4) (°C)**	0.201 ± 0.020	0.158 ± 0.021	0.155
**SD1 (k = 5) (°C)**	0.223 ± 0.022	0.171 ± 0.023	0.122
**SD1 (k = 6) (°C)**	0.240 ± 0.025	0.183 ± 0.024	0.112
**SD1 (k = 7) (°C)**	0.254 ± 0.027	0.191 ± 0.025	0.102
**SD1 (k = 8) (°C)**	0.267 ± 0.029	0.197 ± 0.027	0.094
**SD1 (k = 9) (°C)**	0.279 ± 0.030	0.205 ± 0.029	0.096
**SD1 (k = 10) (°C)**	0.288 ± 0.031	0.212 ± 0.031	0.100
**SD2 (k = 1) (°C)**	0.530 ± 0.054	0.333 ± 0.042	**0.010**
**SD2 (k = 2) (°C)**	0.511 ± 0.053	0.313 ± 0.040	**0.008**
**SD2 (k = 3) (°C)**	0.494 ± 0.053	0.299 ± 0.039	**0.008**
**SD2 (k = 4) (°C)**	0.479 ± 0.052	0.287 ± 0.038	**0.009**
**SD2 (k = 5) (°C)**	0.464 ± 0.052	0.278 ± 0.037	**0.009**
**SD2 (k = 6) (°C)**	0.451 ± 0.051	0.269 ± 0.037	**0.010**
**SD2 (k = 7) (°C)**	0.437 ± 0.049	0.263 ± 0.036	**0.011**
**SD2 (k = 8) (°C)**	0.425 ± 0.049	0.257 ± 0.036	**0.013**
**SD2 (k = 9) (°C)**	0.414 ± 0.048	0.251 ± 0.034	**0.014**
**SD2 (k = 10) (°C)**	0.403 ± 0.047	0.244 ± 0.033	**0.014**

C. 2-hour length time

	Survivors	Non-survivors	p-value
**Number**	23	17	-
**Mean proximal temperature (°C)**	35.08 ± 0.17	35.30 ± 0.25	0.455
**SD of proximal temperature (°C)**	0.351 ± 0.040	0.220 ± 0.036	**0.025**
**SD1 (k = 1) (°C)**	0.078 ± 0.009	0.066 ± 0.013	0.423
**SD1 (k = 2) (°C)**	0.133 ± 0.016	0.111 ± 0.022	0.396
**SD1 (k = 3) (°C)**	0.170 ± 0.019	0.138 ± 0.025	0.307
**SD1 (k = 4) (°C)**	0.199 ± 0.022	0.158 ± 0.027	0.252
**SD1 (k = 5) (°C)**	0.220 ± 0.025	0.171 ± 0.029	0.206
**SD1 (k = 6) (°C)**	0.235 ± 0.027	0.180 ± 0.029	0.182
**SD1 (k = 7) (°C)**	0.246 ± 0.029	0.186 ± 0.029	0.159
**SD1 (k = 8) (°C)**	0.256 ± 0.031	0.190 ± 0.029	0.143
**SD1 (k = 9) (°C)**	0.264 ± 0.032	0.196 ± 0.031	0.147
**SD1 (k = 10) (°C)**	0.269 ± 0.032	0.201 ± 0.033	0.152
**SD2 (k = 1) (°C)**	0.477 ± 0.054	0.288 ± 0.043	**0.014**
**SD2 (k = 2) (°C)**	0.456 ± 0.053	0.266 ± 0.037	**0.010**
**SD2 (k = 3) (°C)**	0.436 ± 0.052	0.251 ± 0.035	**0.006**
**SD2 (k = 4) (°C)**	0.418 ± 0.052	0.239 ± 0.033	**0.006**
**SD2 (k = 5) (°C)**	0.401 ± 0.051	0.230 ± 0.033	**0.007**
**SD2 (k = 6) (°C)**	0.386 ± 0.050	0.222 ± 0.033	**0.015**
**SD2 (k = 7) (°C)**	0.372 ± 0.048	0.216 ± 0.033	**0.017**
**SD2 (k = 8) (°C)**	0.360 ± 0.047	0.209 ± 0.033	**0.020**
**SD2 (k = 9) (°C)**	0.348 ± 0.046	0.201 ± 0.032	**0.019**
**SD2 (k = 10) (°C)**	0.338 ± 0.045	0.193 ± 0.031	**0.018**

D. 1-hour length

	Survivors	Non-survivors	p-value
**Number**	23	17	-
**Mean proximal temperature (°C)**	35.01 ± 0.18	35.26 ± 0.25	0.429
**SD of proximal temperature (°C)**	0.280 ± 0.031	0.189 ± 0.049	0.110
**SD1 (k = 1) (°C)**	0.083 ± 0.012	0.067 ± 0.018	0.427
**SD1 (k = 2) (°C)**	0.139 ± 0.021	0.111 ± 0.030	0.423
**SD1 (k = 3) (°C)**	0.169 ± 0.023	0.134 ± 0.034	0.380
**SD1 (k = 4) (°C)**	0.188 ± 0.025	0.150 ± 0.037	0.379
**SD1 (k = 5) (°C)**	0.196 ± 0.025	0.156 ± 0.038	0.371
**SD1 (k = 6) (°C)**	0.198 ± 0.025	0.159 ± 0.038	0.388
**SD1 (k = 7) (°C)**	0.196 ± 0.026	0.157 ± 0.036	0.376
**SD1 (k = 8) (°C)**	0.195 ± 0.028	0.155 ± 0.034	0.364
**SD1 (k = 9) (°C)**	0.196 ± 0.028	0.156 ± 0.036	0.382
**SD1 (k = 10) (°C)**	0.193 ± 0.027	0.155 ± 0.039	0.412
**SD2 (k = 1) (°C)**	0.358 ± 0.037	0.234 ± 0.055	0.060
**SD2 (k = 2) (°C)**	0.321 ± 0.032	0.204 ± 0.044	**0.034**
**SD2 (k = 3) (°C)**	0.294 ± 0.030	0.185 ± 0.043	**0.039**
**SD2 (k = 4) (°C)**	0.272 ± 0.029	0.173 ± 0.042	**0.049**
**SD2 (k = 5) (°C)**	0.258 ± 0.028	0.167 ± 0.041	0.068
**SD2 (k = 6) (°C)**	0.249 ± 0.028	0.162 ± 0.042	0.086
**SD2 (k = 7) (°C)**	0.242 ± 0.028	0.161 ± 0.045	0.123
**SD2 (k = 8) (°C)**	0.233 ± 0.029	0.160 ± 0.048	0.176
**SD2 (k = 9) (°C)**	0.223 ± 0.031	0.155 ± 0.048	0.227
**SD2 (k = 10) (°C)**	0.217 ± 0.034	0.153 ± 0.049	0.277

E. 30-minute length

	Survivors	Non-survivors	p-value
**Number**	23	17	-
**Mean proximal temperature (°C)**	34.89 ± 0.19	35.20 ± 0.24	0.321
**SD of proximal temperature (°C)**	0.222 ± 0.037	0.189 ± 0.062	0.634
**SD1 (k = 1) (°C)**	0.086 ± 0.016	0.077 ± 0.026	0.761
**SD1 (k = 2) (°C)**	0.140 ± 0.028	0.128 ± 0.046	0.825
**SD1 (k = 3) (°C)**	0.161 ± 0.032	0.151 ± 0.053	0.861
**SD1 (k = 4) (°C)**	0.169 ± 0.036	0.161 ± 0.059	0.893
**SD1 (k = 5) (°C)**	0.167 ± 0.038	0.157 ± 0.064	0.895
**SD1 (k = 6) (°C)**	0.149 ± 0.038	0.152 ± 0.066	0.974
**SD1 (k = 7) (°C)**	0.127 ± 0.039	0.136 ± 0.063	0.900
**SD1 (k = 8) (°C)**	0.086 ± 0.029	0.094 ± 0.041	0.867
**SD2 (k = 1) (°C)**	0.268 ± 0.041	0.222 ± 0.069	0.548
**SD2 (k = 2) (°C)**	0.222 ± 0.032	0.180 ± 0.053	0.482
**SD2 (k = 3) (°C)**	0.192 ± 0.032	0.160 ± 0.052	0.584
**SD2 (k = 4) (°C)**	0.170 ± 0.032	0.151 ± 0.051	0.740
**SD2 (k = 5) (°C)**	0.155 ± 0.034	0.149 ± 0.053	0.914
**SD2 (k = 6) (°C)**	0.147 ± 0.037	0.136 ± 0.054	0.873
**SD2 (k = 7) (°C)**	0.132 ± 0.039	0.121 ± 0.059	0.871
**SD2 (k = 8) (°C)**	0.102 ± 0.029	0.091 ± 0.048	0.843

Appendix 2. Comparison between proximal skin temperature variability indices between survivors and non-survivors at different time-series length (from 1 to 3 hours) during asleep phase.

A. 3-hour length (sleep phase)

	Survivors	Non-survivors	p-value
**Number**	23	17	-
**Mean proximal temperature (°C)**	35.34 ± 0.17	35.56 ± 0.22	0.429
**SD of proximal temperature (°C)**	0.393 ± 0.039	0.284 ± 0.043	0.071
**SD1 (k = 1) (°C)**	0.078 ± 0.005	0.060 ± 0.005	**0.019**
**SD1 (k = 2) (°C)**	0.150 ± 0.014	0.142 ± 0.031	0.806
**SD1 (k = 3) (°C)**	0.178 ± 0.014	0.126 ± 0.011	**0.008**
**SD1 (k = 4) (°C)**	0.222 ± 0.017	0.180 ± 0.028	0.189
**SD1 (k = 5) (°C)**	0.234 ± 0.020	0.159 ± 0.015	**0.007**
**SD1 (k = 6) (°C)**	0.262 ± 0.021	0.199 ± 0.027	0.070
**SD1 (k = 7) (°C)**	0.268 ± 0.024	0.181 ± 0.017	**0.006**
**SD1 (k = 8) (°C)**	0.288 ± 0.025	0.211 ± 0.026	**0.043**
**SD1 (k = 9) (°C)**	0.289 ± 0.027	0.193 ± 0.019	**0.006**
**SD1 (k = 10) (°C)**	0.305 ± 0.028	0.215 ± 0.024	**0.025**
**SD2 (k = 1) (°C)**	0.535 ± 0.057	0.369 ± 0.061	0.059
**SD2 (k = 2) (°C)**	0.525 ± 0.055	0.372 ± 0.060	0.071
**SD2 (k = 3) (°C)**	0.499 ± 0.057	0.348 ± 0.060	0.080
**SD2 (k = 4) (°C)**	0.488 ± 0.054	0.348 ± 0.058	0.091
**SD2 (k = 5) (°C)**	0.464 ± 0.055	0.333 ± 0.058	0.118
**SD2 (k = 6) (°C)**	0.457 ± 0.053	0.331 ± 0.057	0.116
**SD2 (k = 7) (°C)**	0.437 ± 0.053	0.321 ± 0.057	0.151
**SD2 (k = 8) (°C)**	0.432 ± 0.050	0.316 ± 0.055	0.129
**SD2 (k = 9) (°C)**	0.415 ± 0.050	0.312 ± 0.055	0.180
**SD2 (k = 10) (°C)**	0.412 ± 0.048	0.304 ± 0.052	0.140

B. 2-hour length (sleep phase)

	Survivors	Non-survivors	p-value
**Number**	23	17	-
**Mean proximal temperature (°C)**	35.30 ± 0.19	35.54 ± 0.22	0.410
**SD of proximal temperature (°C)**	0.350 ± 0.042	0.245 ± 0.039	0.082
**SD1 (k = 1) (°C)**	0.075 ± 0.006	0.061 ± 0.006	0.094
**SD1 (k = 2) (°C)**	0.146 ± 0.016	0.128 ± 0.022	0.516
**SD1 (k = 3) (°C)**	0.168 ± 0.016	0.128 ± 0.012	0.068
**SD1 (k = 4) (°C)**	0.211 ± 0.021	0.166 ± 0.021	0.144
**SD1 (k = 5) (°C)**	0.219 ± 0.023	0.158 ± 0.016	**0.034**
**SD1 (k = 6) (°C)**	0.248 ± 0.025	0.182 ± 0.021	0.061
**SD1 (k = 7) (°C)**	0.247 ± 0.026	0.175 ± 0.018	**0.031**
**SD1 (k = 8) (°C)**	0.266 ± 0.027	0.190 ± 0.021	**0.033**
**SD1 (k = 9) (°C)**	0.260 ± 0.028	0.184 ± 0.019	**0.033**
**SD1 (k = 10) (°C)**	0.273 ± 0.028	0.194 ± 0.021	**0.028**
**SD2 (k = 1) (°C)**	0.470 ± 0.060	0.323 ± 0.056	0.092
**SD2 (k = 2) (°C)**	0.462 ± 0.058	0.314 ± 0.054	0.079
**SD2 (k = 3) (°C)**	0.430 ± 0.059	0.295 ± 0.054	0.113
**SD2 (k = 4) (°C)**	0.420 ± 0.056	0.286 ± 0.053	0.099
**SD2 (k = 5) (°C)**	0.389 ± 0.057	0.275 ± 0.052	0.162
**SD2 (k = 6) (°C)**	0.385 ± 0.054	0.269 ± 0.050	0.135
**SD2 (k = 7) (°C)**	0.361 ± 0.054	0.263 ± 0.049	0.203
**SD2 (k = 8) (°C)**	0.364 ± 0.050	0.258 ± 0.047	0.144
**SD2 (k = 9) (°C)**	0.341 ± 0.049	0.253 ± 0.045	0.214
**SD2 (k = 10) (°C)**	0.342 ± 0.045	0.245 ± 0.043	0.141

C. 1-hour length (sleep phase)

	Survivors	Non-survivors	p-value
**Number**	23	17	-
**Mean proximal temperature (°C)**	35.25 ± 0.20	35.49 ± 0.20	0.419
**SD of proximal temperature (°C)**	0.235 ± 0.028	0.174 ± 0.030	0.154
**SD1 (k = 1) (°C)**	0.068 ± 0.006	0.058 ± 0.006	0.280
**SD1 (k = 2) (°C)**	0.117 ± 0.012	0.096 ± 0.012	0.227
**SD1 (k = 3) (°C)**	0.147 ± 0.017	0.119 ± 0.017	0.256
**SD1 (k = 4) (°C)**	0.166 ± 0.020	0.135 ± 0.021	0.303
**SD1 (k = 5) (°C)**	0.176 ± 0.023	0.142 ± 0.025	0.330
**SD1 (k = 6) (°C)**	0.179 ± 0.024	0.147 ± 0.028	0.386
**SD1 (k = 7) (°C)**	0.178 ± 0.025	0.152 ± 0.030	0.507
**SD1 (k = 8) (°C)**	0.174 ± 0.025	0.154 ± 0.031	0.610
**SD1 (k = 9) (°C)**	0.169 ± 0.025	0.151 ± 0.031	0.648
**SD1 (k = 10) (°C)**	0.160 ± 0.024	0.145 ± 0.029	0.687
**SD2 (k = 1) (°C)**	0.314 ± 0.038	0.232 ± 0.042	0.162
**SD2 (k = 2) (°C)**	0.287 ± 0.035	0.213 ± 0.041	0.177
**SD2 (k = 3) (°C)**	0.259 ± 0.032	0.193 ± 0.039	0.193
**SD2 (k = 4) (°C)**	0.232 ± 0.027	0.177 ± 0.036	0.223
**SD2 (k = 5) (°C)**	0.213 ± 0.023	0.166 ± 0.032	0.228
**SD2 (k = 6) (°C)**	0.201 ± 0.021	0.154 ± 0.028	0.181
**SD2 (k = 7) (°C)**	0.197 ± 0.023	0.142 ± 0.023	0.103
**SD2 (k = 8) (°C)**	0.196 ± 0.025	0.134 ± 0.020	0.076
**SD2 (k = 9) (°C)**	0.196 ± 0.028	0.129 ± 0.020	0.076
**SD2 (k = 10) (°C)**	0.196 ± 0.031	0.125 ± 0.019	0.081

Appendix 3. Predictive effect of proximal temperature variability (PTV) indices on one-year mortality: A. Univariate analysis, B. Bivariate analysis to assess the independence of PTV parameters from markers of liver failure (MELD) in predicting mortality.

A1. Univariate analysis on 6-hour length time-series

	β	Hazard Ratio	95% CI	p-value
**SD of proximal temperature (°C)**	-3.055	0.047	0.002, 0.922	**0.044**
**SD1 (k = 3) (°C)**	-11.742	0.000	0.000, 0.257	**0.027**
**SD1 (k = 4) (°C)**	-10.207	0.000	0.000, 0.210	**0.021**
**SD1 (k = 5) (°C)**	-9.053	0.000	0.000, 0.213	**0.018**
**SD1 (k = 6) (°C)**	-8.122	0.000	0.000, 0.237	**0.017**
**SD1 (k = 7) (°C)**	-7.296	0.001	0.000, 0.271	**0.017**
**SD1 (k = 8) (°C)**	-6.463	0.002	0.000, 0.335	**0.018**
**SD1 (k = 9) (°C)**	-5.840	0.003	0.000, 0.393	**0.020**
**SD1 (k = 10) (°C)**	-5.540	0.004	0.000, 0.408	**0.019**
**SD2 (k = 1) (°C)**	-2.199	0.111	0.013, 0.930	**0.043**
**SD2 (k = 2) (°C)**	-2.180	0.113	0.013, 0.951	**0.045**
**SD2 (k = 3) (°C)**	-2.143	0.117	0.014, 0.991	**0.049**
**SD2 (k = 4) (°C)**	-2.130	0.119	0.014, 1.026	0.053
**SD2 (k = 5) (°C)**	-2.110	0.121	0.014, 1.068	0.057
**SD2 (k = 6) (°C)**	-2.081	0.125	0.014, 1.121	0.063
**SD2 (k = 7) (°C)**	-2.058	0.128	0.014, 1.181	0.070
**SD2 (k = 8) (°C)**	-2.056	0.128	0.013, 1.232	0.075
**SD2 (k = 9) (°C)**	-2.069	0.126	0.013, 1.276	0.080
**SD2 (k = 10) (°C)**	-2.066	0.127	0.012, 1.330	0.085

B1. Bivariate analysis on 6-hour length time-series

	β	Hazard Ratio	95% CI	p-value
**SD of proximal temperature (°C)**	-3.338	0.036	0.002, 0.595	**0.020**
**SD1 (k = 3) (°C)**	-10.861	0.000	0.000, 0.431	**0.034**
**SD1 (k = 4) (°C)**	-9.590	0.000	0.000, 0.269	**0.023**
**SD1 (k = 5) (°C)**	-8.706	0.000	0.000, 0.220	**0.018**
**SD1 (k = 6) (°C)**	-8.050	0.000	0.000, 0.211	**0.015**
**SD1 (k = 7) (°C)**	-7.477	0.001	0.000, 0.216	**0.014**
**SD1 (k = 8) (°C)**	-6.873	0.001	0.000, 0.247	**0.014**
**SD1 (k = 9) (°C)**	-6.274	0.002	0.000, 0.297	**0.015**
**SD1 (k = 10) (°C)**	-5.755	0.003	0.000, 0.358	**0.017**
**SD2 (k = 1) (°C)**	-2.419	0.089	0.012, 0.670	**0.019**
**SD2 (k = 2) (°C)**	-2.437	0.087	0.011, 0.668	**0.019**
**SD2 (k = 3) (°C)**	-2.426	0.088	0.011, 0.681	**0.020**

A2. Univariate analysis on 3-hour length time-series

	β	Hazard Ratio	95% CI	p-value
**SD of proximal temperature (°C)**	-3.576	0.028	0.001, 0.660	**0.027**
**SD2 (k = 1) (°C)**	-2.743	0.064	0.006, 0.663	**0.021**
**SD2 (k = 2) (°C)**	-2.902	0.055	0.005, 0.612	**0.018**
**SD2 (k = 3) (°C)**	-2.950	0.052	0.004, 0.610	**0.019**
**SD2 (k = 4) (°C)**	-2.994	0.050	0.004, 0.613	**0.019**
**SD2 (k = 5) (°C)**	-3.037	0.048	0.004, 0.620	**0.020**
**SD2 (k = 6) (°C)**	-3.094	0.045	0.003, 0.626	**0.021**
**SD2 (k = 7) (°C)**	-3.141	0.043	0.003, 0.646	**0.023**
**SD2 (k = 8) (°C)**	-3.185	0.041	0.003, 0.673	**0.025**
**SD2 (k = 9) (°C)**	-3.274	0.038	0.002, 0.685	**0.027**
**SD2 (k = 10) (°C)**	-3.382	0.034	0.002, 0.693	**0.028**

B2. Bivariate analysis on 3-hour length time-series

	β	Hazard Ratio	95% CI	p-value
**SD of proximal temperature (°C)**	-3.798	0.022	0.001, 0.668	**0.028**
**SD2 (k = 1) (°C)**	-2.933	0.053	0.004, 0.649	**0.022**
**SD2 (k = 2) (°C)**	-3.119	0.044	0.003, 0.588	**0.018**
**SD2 (k = 3) (°C)**	-3.183	0.041	0.003, 0.583	**0.018**
**SD2 (k = 4) (°C)**	-3.259	0.038	0.003, 0.575	**0.018**
**SD2 (k = 5) (°C)**	-3.335	0.036	0.003, 0.572	**0.019**
**SD2 (k = 6) (°C)**	-3.400	0.033	0.002, 0.574	**0.019**
**SD2 (k = 7) (°C)**	-3.436	0.032	0.002, 0.595	**0.021**
**SD2 (k = 8) (°C)**	-3.499	0.030	0.001, 0.617	**0.023**
**SD2 (k = 9) (°C)**	-3.622	0.027	0.001, 0.627	**0.024**
**SD2 (k = 10) (°C)**	-3.744	0.024	0.001, 0.643	**0.026**

A3. Univariate analysis on 2-hour length time-series

	β	Hazard Ratio	95% CI	p-value
**SD of proximal temperature (°C)**	-3.709	0.025	0.001, 0.845	**0.040**
**SD2 (k = 1) (°C)**	-2.983	0.051	0.004, 0.709	**0.027**
**SD2 (k = 2) (°C)**	-3.326	0.036	0.002, 0.594	**0.020**
**SD2 (k = 3) (°C)**	-3.428	0.032	0.002, 0.590	**0.021**
**SD2 (k = 4) (°C)**	-3.481	0.031	0.002, 0.611	**0.022**
**SD2 (k = 5) (°C)**	-3.495	0.030	0.001, 0.655	**0.026**
**SD2 (k = 6) (°C)**	-3.541	0.029	0.001, 0.698	**0.029**
**SD2 (k = 7) (°C)**	-3.560	0.028	0.001, 0.765	**0.034**
**SD2 (k = 8) (°C)**	-3.585	0.028	0.001, 0.826	**0.038**
**SD2 (k = 9) (°C)**	-3.734	0.024	0.001, 0.831	**0.039**
**SD2 (k = 10) (°C)**	-3.933	0.020	0.000, 0.827	**0.039**

B3. Bivariate analysis on 2-hour length time-series

	β	Hazard Ratio	95% CI	p-value
**SD of proximal temperature (°C)**	-3.578	0.028	0.001, 1.288	0.067
**SD2 (k = 1) (°C)**	-2.900	0.055	0.003, 0.948	**0.046**
**SD2 (k = 2) (°C)**	-3.225	0.040	0.002, 0.819	**0.037**
**SD2 (k = 3) (°C)**	-3.291	0.037	0.002, 0.861	**0.040**
**SD2 (k = 4) (°C)**	-3.334	0.036	0.001, 0.928	**0.045**
**SD2 (k = 5) (°C)**	-3.347	0.035	0.001, 1.027	0.052
**SD2 (k = 6) (°C)**	-3.414	0.033	0.001, 1.099	0.056
**SD2 (k = 7) (°C)**	-3.451	0.032	0.001, 1.201	0.063
**SD2 (k = 8) (°C)**	-3.523	0.030	0.001, 1.274	0.067
**SD2 (k = 9) (°C)**	-3.716	0.024	0.000, 1.262	0.065
**SD2 (k = 10) (°C)**	-3.879	0.021	0.000, 1.284	0.066

A4. Univariate analysis on 1-hour length time-series

	β	Hazard Ratio	95% CI	p-value
**SD2 (k = 2) (°C)**	-4.263	0.014	0.000, 0.679	**0.031**
**SD2 (k = 3) (°C)**	-4.628	0.010	0.000, 0.697	**0.034**
**SD2 (k = 4) (°C)**	-4.737	0.009	0.000, 0.825	**0.041**

B3. Bivariate analysis on 1-hour length time-series

	β	Hazard Ratio	95% CI	p-value
**SD2 (k = 2) (°C)**	-4.082	0.017	0.000, 0.808	**0.039**
**SD2 (k = 3) (°C)**	-4.298	0.014	0.000, 0.919	**0.046**
**SD2 (k = 4) (°C)**	-4.419	0.012	0.000, 1.064	0.053

Appendix 4. Predictive effect of proximal temperature variability (PTV) indices calculated from the sleep phase on one-year mortality: A. Univariate analysis, B. Bivariate analysis to assess the independence of PTV parameters from markers of liver failure (MELD) in predicting mortality.

A1. Univariate analysis on 3-hour length time-series (sleep phase)

	β	Hazard Ratio	95% CI	p-value
**SD1 (k = 1) (°C)**	-28.983	0.000	0.000, 0.003	**0.014**
**SD1 (k = 3) (°C)**	-12.671	0.000	0.000, 0.039	**0.008**
**SD1 (k = 5) (°C)**	-9.155	0.000	0.000, 0.108	**0.010**
**SD1 (k = 7) (°C)**	-7.688	0.000	0.000, 0.187	**0.012**
**SD1 (k = 8) (°C)**	-5.127	0.006	0.000, 0.828	**0.042**
**SD1 (k = 9) (°C)**	-6.980	0.001	0.000, 0.240	**0.014**
**SD1 (k = 10) (°C)**	-5.364	0.005	0.000, 0.584	**0.029**

B1. Multivariate analysis on 3-hour length time-series (sleep phase)

	β	Hazard Ratio	95% CI	p-value
**SD1 (k = 1) (°C)**	-28.295	0.000	0.000, 0.001	**0.010**
**SD1 (k = 3) (°C)**	-13.298	0.000	0.000, 0.010	**0.003**
**SD1 (k = 5) (°C)**	-10.442	0.000	0.000, 0.021	**0.002**
**SD1 (k = 7) (°C)**	-9.078	0.000	0.000, 0.036	**0.002**
**SD1 (k = 8) (°C)**	-6.228	0.002	0.000, 0.264	**0.013**
**SD1 (k = 9) (°C)**	-8.227	0.000	0.000, 0.057	**0.003**
**SD1 (k = 10) (°C)**	-6.195	0.002	0.000, 0.231	**0.010**

A2. Univariate analysis on 2-hour length time-series (sleep phase)

	β	Hazard Ratio	95% CI	p-value
**SD1 (k = 5) (°C)**	-6.216	0.002	0.000, 0.990	0.050
**SD1 (k = 7) (°C)**	-5.408	0.004	0.000, 0.924	**0.047**
**SD1 (k = 8) (°C)**	-4.807	0.008	0.000, 0.952	**0.048**
**SD1 (k = 9) (°C)**	-4.844	0.008	0.000, 1.033	0.052
**SD1 (k = 10) (°C)**	-4.790	0.008	0.000, 0.915	**0.046**

B2. Multivariate analysis on 2-hour length time-series (sleep phase)

	β	Hazard Ratio	95% CI	p-value
**SD1 (k = 7) (°C)**	-6.117	0.002	0.000, 0.379	**0.020**
**SD1 (k = 8) (°C)**	-5.220	0.005	0.000, 0.550	**0.027**
**SD1 (k = 10) (°C)**	-5.024	0.007	0.000, 0.653	**0.032**
